# Active promoters give rise to false positive ‘Phantom Peaks’ in ChIP-seq experiments

**DOI:** 10.1093/nar/gkv637

**Published:** 2015-06-27

**Authors:** Dhawal Jain, Sandro Baldi, Angelika Zabel, Tobias Straub, Peter B. Becker

**Affiliations:** Biomedical Center and Center for Integrated Protein Science Munich, Ludwig-Maximilians-University, Munich, Germany

## Abstract

Chromatin immunoprecipitation (ChIP) is widely used to identify chromosomal binding sites. Chromatin proteins are cross-linked to their target sequences in living cells. The purified chromatin is sheared and the relevant protein is enriched by immunoprecipitation with specific antibodies. The co-purifying genomic DNA is then determined by massive parallel sequencing (ChIP-seq).

We applied ChIP-seq to map the chromosomal binding sites for two ISWI-containing nucleosome remodeling factors, ACF and RSF, in *Drosophila* embryos. Employing several polyclonal and monoclonal antibodies directed against their signature subunits, ACF1 and RSF-1, robust profiles were obtained indicating that both remodelers co-occupied a large set of active promoters.

Further validation included controls using chromatin of mutant embryos that do not express ACF1 or RSF-1. Surprisingly, the ChIP-seq profiles were unchanged, suggesting that they were not due to specific immunoprecipitation. Conservative analysis lists about 3000 chromosomal loci, mostly active promoters that are prone to non-specific enrichment in ChIP and appear as ‘Phantom Peaks’. These peaks are not obtained with pre-immune serum and are not prominent in input chromatin.

Mining the modENCODE ChIP-seq profiles identifies potential Phantom Peaks in many profiles of epigenetic regulators. These profiles and other ChIP-seq data featuring prominent Phantom Peaks must be validated with chromatin from cells in which the protein of interest has been depleted.

## INTRODUCTION

The validity, quality and robustness of ChIP-seq experiments depends on several variables, such as the specificity and avidity of the antibodies used, the fractional occupancy of chromatin loci by the protein of interest, the nature of its chromatin interaction and the likelihood that this interaction will be trapped by chemical crosslinking. Furthermore, native binding profiles may be distorted by biases introduced by experimental procedures, including the immunoprecipitation process, the shearing of chromatin, DNA library preparation, sequencing and the final bioinformatics analysis (1). To avoid scoring false positive signals the analysis of control libraries obtained from input chromatin and ‘mock’ ChIP reactions, in which the specific antibody is omitted, are currently recommended (2,3).

Nucleosome remodeling factors of the ISWI-type are able to slide nucleosomes on DNA to either expose DNA sequences or to close gaps in the nucleosome fiber (4,5). *Drosophila* RSF, ACF and CHRAC (and their orthologous complexes in yeast) are best known for their role in regenerating the integrity of the nucleosomal arrays (nucleosome ‘spacing’) after inevitable disruptions of the fiber during utilization of the genetic information (6–13).

These remodelers presumably interact rather transiently with chromatin and thus are difficult to trap using ChIP-based mapping (14). Indeed, it has been estimated that ∼90% of the large pool of ISWI molecules in human cells are not chromatin-bound in steady state (15,16). Nevertheless, chromatin interaction profiles of *Drosophila* ISWI have been described, although, perhaps due to the fact that different tissues were analyzed, they do not agree particularly well with each other (17,18). Recently, using a highly sensitive ChIP-exo method that eliminates background signals, the yeast Isw1 and Isw2 complexes were found associated with promoter-proximal nucleosomes (19). Furthermore, the mouse ISWI orthologue SNF2h has been found co-localized with the remodeling ATPases Brg1 and Chd4 at many loci, suggesting extensive cooperation of remodelers in generating access to regulatory sequences (20).

Encouraged by these findings we aimed at defining the binding sites of three ISWI containing ATP-dependent nucleosome remodeling factors CHRAC, ACF and RSF (4,9,21) in *Drosophila* embryos. To distinguish these factors from other ISWI-containing remodelers, we employed antibodies directed against their signature subunits, ACF1 and RSF-1. We obtained robust and high-quality ChIP-seq profiles by all accepted modENCODE standards. The excellent signal to noise ChIP-seq profiles suggested co-localization of the two remodeling factors at the active promoter regions. However, upon further scrutiny we had to realize that we encountered a hitherto unappreciated type of prominent false positive signal, which potentially affects the quality of many ChIP-seq profiles beyond our study. Our inventory of Phantom Peak loci may help to scrutinize ChIP-seq profiles for potential artifacts. Signals that coincide with Phantom Peak sites should be interpreted with caution and subjected to further validation.

## MATERIALS AND METHODS

### Chromatin immunoprecipitation and sequencing

We used 0–12 h old Oregon-R wild-type (WT) embryos for chromatin immunoprecipitation (ChIP) assays. The mutant alleles *acf1^7^* (manuscript in preparation) and *rsf-1^3602^* (9) were generated by imprecise P-element excisions. Unstaged embryos (1 g) were dechorinated in 120 ml, 1:5 diluted, sodium hypochloride (VWR, Cat.no. 301696S) for 3 min. The embryos were thoroughly washed and fixed in the fixing solution [10 ml, 0.1 M NaCl, 0.05 M HEPES pH 8.0, 1 mM EDTA, 0.5 mM EGTA, 3.7% Formaldehyde (Sigma, Cat. No F1635) added to 30 ml n-Heptane (VWR, Cat. No. 8.22332.1000)] for 15 min at 16–18°C on a rotating wheel. Fixation was quenched by adding 125 mM glycine. The embryos were subsequently washed with phosphate buffered saline (including 0.01% Triton-X100) for 10 min and stored at −80°C until further use.

For nuclei isolation, embryos were slowly thawed and dounced using a glass homogenizer (Schubert, Cat.no. 9164693) with 20 strokes each of the A and B pestles in ice-cold NX-I buffer [15 mM HEPES pH 7.6, 10 mM KCl, 2 mM MgCl2, 0.5 mM EGTA, 0.1 mM EDTA, 350 mM sucrose, 1 mM DTT, 0.2 mM PMSF, Protease inhibitors Leupeptin, Pepstatin and Aprotinin (10 μg/ml)]. The lysate was filtered through Miracloth and nuclei were pelleted at 3500 rpm, 10 min, 4°C. The pellet was washed in NX-I buffer. Finally, the nuclei were resuspended in RIPA [1% Triton X-100, 0.1% Sodium deoxycholate, 140 mM NaCl, 10 mM Tris pH 8.0, 1 mM EDTA, 0.1% SDS, 1 mM PMSF] and washed three times. Nuclei were then counted and frozen at −80°C in aliquots of ∼10^9^ nuclei/ml.

For shearing and ChIP, thawed nuclei were adjusted to ∼2×10^8^/ml by dilution in RIPA and sheared with a Covaris S220 system (Covaris Inc. MA, USA) at 110 Watts, 20% duty factor and 200 cycles per burst for 25 min. Chromatin was pre-cleared using protein a A+G bead (1:1) mix for 1h at 4°C. Immunoprecipitations were set up overnight at 4°C with 200 μl chromatin and 4 μl of the respective antibody adjusted to 500 μl with RIPA. The antibody amounts had been titrated by following ChIP-qPCR enrichment along several candidate regions obtained from modENCODE ISWI profiles (Supplementary Figure S2A, data not shown). RIPA-equilibrated protein A+G (1:1) mix was then added to precipitate the immune-complexes for 3 h at 4°C. For the rat monoclonal antibody (3F1), the chromatin immunoprecipitation was performed using pre-sorbed protein G beads, with an excess of antibody, for 3 h at 4°C. Beads were washed subsequently five times for 10 min each in 1 ml RIPA buffer. Residual RNA was digested by RNase A (10 μg/100 μl, Sigma, Cat. No. R4875) at 37°C for 20 min. Subsequent protein digestion (25 μg/100 μl, Proteinase K, Genaxxon, Cat.no. M3036.0100) and reversal of cross-linking were performed together at 68°C for 2 h. DNA was purified using GenElute™ PCR Clean-Up Kit (Sigma, Cat.no NA1020).

ChIP DNA was quantified using the Qubit® dsDNA HS Assay Kit (Life Technologies, Cat.no.Q32851) and sequencing libraries were prepared using the MicroPlex Library Preparation kit (Diagenode, Cat. No. C05010011) starting from 2 ng DNA, whenever possible. PCR amplification was monitored by quantifying amplified libraries (maximum 19 cycles). The libraries were sequenced on a HighSeq 1500 (Illumina) instrument to yield roughly 15–25 Mio reads of 50 bp, single end sequences per sample. Raw, de-multiplexed sequence data files are available at GEO (GSE67323).

### Antibodies and immunological procedures

The rabbit polyclonal antibodies Rb1 and Rb2 were raised against ACF1 amino acids (aa) 1065–1463, while the rat monoclonal antibody 3F1 was raised against full length ACF1 but recognizes an epitope in the N-terminal half (data not shown). The rabbit polyclonal antibody was raised against RSF-1 aa 2049–2390. For Western blotting, an overnight collection of 100–300 mg *Drosophila* embryos of *acf1^7^, rsf-1^3602^* and WT genotypes were used for nuclei preparation as described earlier in the method section. Nuclear extract was prepared using nuclear complex co-IP kit (Active Motif, Cat.no. 54001) according to manufacturer's guidelines. Immunofluorescence microscopy analyses were carried as described (22) using a Leica TCS SP5 II (Leica Microsystems Inc.) and a 20X objective.

### Analysis of sequencing data

Raw reads were mapped to *Drosophila melanogaster* genome assembly Dmel3 v5.75 using Bowtie v1.1.1 with unique mapping criteria of ‘m-1’. The quality of the raw reads was assessed using FASTQC v0.10.1. Background-subtracted tag densities were obtained using the SPP package (23).

Peak, motif identification and peak annotation were performed using the HOMER suite, v4.7 under default parameter settings (24). Sequencing tracks were visualized by IGB (25) and IGV (26) genome viewers. All subsequent analyses of data comparison with modENCODE data sets, DamID-chip data sets (27,28) or other publically available profiles were done using custom written R scripts and Bioconductor packages (ChIPPeaksAnno, DESeq2 and Venneuler) (29,30). Coverage objects for the ChIP and input samples were generated by size-normalizing the libraries to 1 Mio reads. For DamID data sets, the coordinates of the tags from GPL8471 platform were remapped on Dmel3 v5.75 using GMAP (31). Potential factor binding sites in the DamID data sets were derived by identifying top 1% scoring tags on the chip. The tags were then subsequently extended to 1 kb and overlapping intervals were merged.

## RESULTS

### ChIP-seq mapping of ACF1 and RSF-1 suggests chromosomal co-localisation

For ChIP we made use of two different rabbit polyclonal antibodies (Rb1, Rb2) raised against the C-terminal region of ACF1, a rat monoclonal antibody (3F1) that detects the N-terminus of ACF1 and a rabbit polyclonal antibody raised against a central fragment of RSF-1. The specificities of the antibodies were assessed by Western blotting and immunofluorescence microscopy (IFM) of mutant embryos. *acf1^7^* and *rsf-1^3602^* mutants had previously been generated by P-element integration into the 5′ ends of the coding sequences, followed by imprecise excision leading to deletions (9). The *acf1^7^* mutation deletes the genomic region of the ACF1 gene encoding aa 41 – 951 (manuscript in preparation). The deletions are documented by the absence of sequencing reads for the corresponding genomic region in input chromatin of the *acf1^7^* mutant sample and absence of traces obtained from RNA polymerase II ChIP-seq enrichments over the gene body in the *rsf-1^3602^* background (Supplementary Figure S1A, B). The absence of ACF1 or RSF-1 expression in mutant embryos was confirmed on Western blots and by IFM (Supplementary Figure S1C, D). The ACF1 monoclonal antibody 3F1 and the polyclonal Rb1 are highly specific, whereas the Rb2 shows minor cross-reactivity with some extract proteins (Supplementary Figure S1C). The RSF-1 antibody, which allows detection of the antigen in *Drosophila* embryos by IFM, does not yield signal in RSF-1-deficient embryos, demonstrating its specificity (Supplementary Figure S1D).

Formaldehyde-fixed chromatin from embryos collected during a 12 h time window (0–12 h after egg laying) was sheared with adaptive focused acoustics (Covaris) to 200 base pair (bp) mean fragments and subjected to ChIP-seq analysis (for details, see Materials and Methods). When tested on candidate genomic loci obtained from modENCODE ISWI profiles (modE3030, 3031, 3032 and 5062) all antibodies show enrichment of the corresponding amplicons by quantitative PCR (Supplementary Figure S2A, Supplementary Table S1). The antibodies were titrated in the dilution range of 1:62.5–1:1000 and a 1:125 dilution (4 μl of rabbit antiserum) was selected for the immunoprecipitation. This antibody concentration is comparable to our previous ChIP studies where we monitored JIL-1 (5 μl antiserum), MSL1 (5 μl), MSL2 (1 μl), MSL3 (4 μl), MLE (4 μl) and MOF (2.5 μl) and obtained meaningful profiles of excellent quality. The RSF-1 antibody works under similar concentration range in both ChIP and IFM (1:150 dilution) (Supplementary Figure S1D).

**Figure 1.**
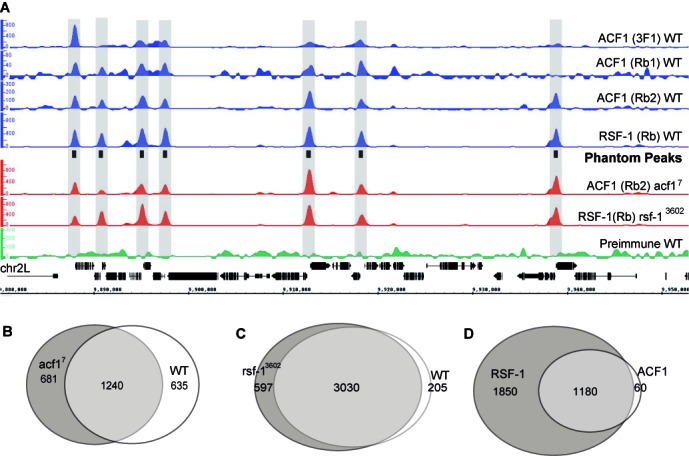
ChIP-Seq profiles obtained for ACF1 and RSF-1 in wild-type and mutant embryos show strong overlap. (**A**) Smoothed and background-subtracted tag density profiles are displayed over a representative region of chromosome 2L. The profiles were obtained with antibodies directed against ACF1 or RSF-1 by ChIP from chromatin of wild-type (WT) and mutant embryos as indicated to the right. The positions of Phantom Peaks are indicated by black boxes in the center track and gray-shaded rectangles across all profiles. (**B**, **C**) Venn diagrams illustrating the overlap between the peak regions of (B) ACF1 in WT and *acf1^7^* mutant embryos and (C) RSF-1 in WT and *rsf-1^3602^* mutant embryos. The peaks overlapping in (B) or (C) were termed ‘ACF1 common’ or ‘RSF-1 common’, respectively. (**D**) Venn diagram illustrating the overlap between ‘ACF1 common’ and ‘RSF-1 common’ peaks. The union of those peaks yields a set of 3090 loci, which we term ‘Phantom Peaks’.

The smoothened, background-subtracted sequence tag density profiles are all highly similar and reveal series of prominent peaks, albeit with variable intensities between different antibodies (Figure 1A, Supplementary Figure S3). For ACF1, the monoclonal antibody shows the best signal-to-noise ratio, but this is dwarfed by the very intense RSF-1 signals. Peaks derived using the polyclonal ACF1 antibodies coincided well with the larger list obtained with the monoclonal antibody 3F1 (Supplementary Figure S2B). The good coincidence of ACF1 and RSF-1 profiles with well-defined peaks suggested co-localization predominantly at the nucleosome-free regions in gene promoters.

### Phantom Peaks

The selective interaction of both remodelers mainly with promoters was unexpected given the presumed role of CHRAC/ACF and RSF as general chromatin organizers. To gain further confidence in the ChIP-seq profiles we repeated the analysis with *acf1^7^* and *rsf-1^3602^* mutant embryos that do not express the respective factors. Surprisingly, we obtained nearly identical genomic ChIP-seq enrichments (Figure 1A, Supplementary Figure S3). In the absence of antigen the peak profiles cannot be due to specific chromatin immunoprecipitation and hence must be considered ‘false positive’.

We tried to rule out trivial explanations for this result. These peaks are not prominent in input chromatin, ruling out a bias generated by selective solubilisation of accessible sites. The different parts of ACF1 and RSF-1 that were used for immunization do not show amino acid sequence similarity. Therefore, it is unlikely that each individual serum enriched shared cross-reactive promoter factor or show cross-reactivity toward the other analyzed remodeling factor. Moreover, ‘bead-only’ ChIP reactions, in which the primary antibody was omitted or using pre-immune serum, did not enrich these regions (Figure 1A, Supplementary Figure S3, data not shown). Curiously, these peaks, which we will refer to as ‘Phantom Peaks’, only appear during ChIP involving real antibodies.

It was previously suggested that unannotated high copy number regions in the genome may lead to erroneous and false positive peak calling (32). Phantom Peaks are not due to this numerical artifact. First, visual inspection of browser screen shots reveals their location at promoters. Second, Phantom Peaks are not enriched in the input samples (Supplementary Figure S4). We further compared Phantom Peak regions to the 30599 regions obtained as the top 1% scoring regions in the input samples (high read density regions, HDRs). These HDRs correspond to ∼3% of the genome. Our analysis shows that only 5% of the Phantom Peaks overlap with the top 1% input signal (*P*-value <0.05) (33) indicating that the peak regions are either enriched during immunoprecipitation stage or selectively amplified during library preparation.

We derived conservative sets of peaks for ACF1 and RSF-1 by employing the HOMER software (24). The profiles (Supplementary Table S2) generated using different antibodies were considered as replicates and a common peak list was obtained for each remodeling factor in wild-type (WT) and mutant background. The HOMER software has the advantage that it is geared to identify peaks of fixed width, an approach that conveys increased sensitivity during the peak calling procedure.

Among the genomic sites enriched with ACF1 antibodies, 1240 regions are shared between WT and *acf1^7^* chromatin profiles (Figure 1B). The uniquely identified ACF1 enrichment regions in WT show persistent ChIP-seq signals enrichment in the *acf1^7^* background, irrespective of whether they are called as peaks or not in the latter (and vice versa, data not shown). Likewise, the avid RSF-1 antibody retrieves 3030 common regions from chromatin of WT and *rsf-1^3602^* embryos (Figure 1C). The vast majority of the ACF1 false positive peaks are also recovered by ChIP with the RSF-1 antibody (Figure 1D). The lists of false positive peaks from either sample were combined to generate a list of 3090 unique peaks that we refer to as ‘Phantom Peaks’ (Supplementary Table S3). The comparison of the replicate ChIP-seq profiles along 3090 Phantom Peaks using Spearmann-ranked correlation suggests a good correlation within the replicate profiles and a clear separation from input samples (Supplementary Figure S2C).

Conceivably, over-fixation of tissue using higher concentrations of formaldehyde (FA) might be a source of artifacts. Our ChIP-seq profiles were derived from 3.7% FA fixed tissues. We previously optimized and used this setting to obtain genome-wide localization of MSL1 (34), which is distinct from Phantom Peaks. However, in order to exclude any potential biases, we repeated the ChIP-seq profile for ACF1 in S2 cells fixed with 1% FA. Two profiles were generated using 3F1 and Rb2 antibodies which are in good agreement with each other. Cross-correlation analysis suggests that these profiles are of high quality, with consensus1652 peaks identified as sites of enrichment. About 77% of these sites overlap with Phantom Peaks. For the additional Phantom Peaks enriched signals can be detected in S2 chromatin profiles by visual inspection or cumulative signal density plotting (Supplementary Figure S5). We also profiled embryos fixed with 1.8% FA, a fixation condition resembling modENCODE protocols for *Drosophila* tissues. The profiles were in good agreement with the embryo ACF1 ChIP-seq profiles obtained from 3.7% FA fixed samples where all the identified peaks overlap with the Phantom Peaks (Supplementary Figure S6). To summarize, Phantom Peaks are not a result of a particular FA fixation protocol.

### Phantom Peaks map predominantly to active promoters

**Figure 2.**
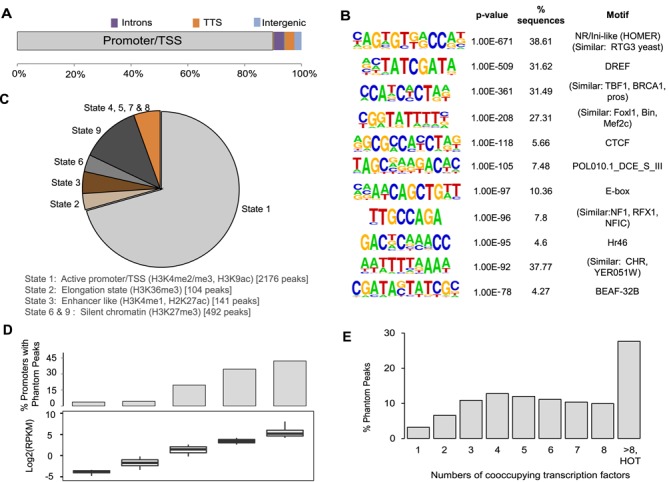
Characterization of the Phantom Peaks. (**A**) Distribution of the 3090 Phantom Peaks between promoters, introns, transcription termination sites (TTS) and intergenic regions. Localization to coding regions or UTRs was negligible. (**B**) Prevalent sequence motifs within Phantom Peak regions. For each motif the sequence logo, its *P*-value of enrichment, the fraction of regions with a motif and the best matching motif in the JASPAR database are indicated. (**C**) Annotation of Phantom Peak localization according to the ‘Nine States’ of chromatin (35). (**D**) The prevalence of Phantom Peaks correlates with promoter strength. Promoters were binned into five equally-sized groups based on the 10–12 h old WT embryo expression data (36). For each group the gene expression levels and the fraction of promoters containing a Phantom Peak are displayed. (**E**) Phantom Peaks tend to map to clusters of transcription factor binding sites. Sites containing the indicated number of transcription factor binding events were derived from modENCODE (37). Sites with more than 8 transcription factor binding events are termed ‘HOT regions’. The graph shows the fractions of Phantom Peaks that harbor the given number of transcription factor binding sites.

The annotation of Phantom Peak localization revealed that about 90% of the peaks fall in a region of 1000 bp around transcription start sites (TSS), identifying promoters as major contributors (Figure 2A). Further 3% of Phantom Peaks localize to transcription termination sites (TTS) and introns. *De novo* DNA sequence motif analysis using HOMER reveals promoter motifs that are enriched in up to 40% of the peaks (Figure 2B). For several highly enriched motifs, the corresponding interacting proteins in *Drosophila* are not known, however these motifs share strong similarities to known recognition motifs for mammalian or yeast proteins. Consensus binding sites for a number of transcription factors and insulator proteins, such as BEAF-32 and CTCF, are also present (Figure 2B, Supplementary Table S4). Most of the genes carrying Phantom Peaks in the promoter regions tend to be involved in either house-keeping processes such as cytoskeletal/ spindle organization, vesicle transport, transcription and RNA metabolism, or are genes expressed during embryonic morphogenesis (data not shown). In line with this annotation, 70% of the peaks map to open chromatin regions derived from S2 cells [state 1 in the modENCODE nomenclature (35)] that are characterized by prevalence of active histone modifications such as H3K4me3 and H3K9ac (Figure 2C). We further annotated the Phantom Peak locations with respect to histone modifications during fly development by employing a binary scoring scheme for overlapping intervals with the modENCODE histone ChIP-seq peaks. Phantom Peaks tend to reside in active chromatin across different developmental stages (Supplementary Figure S7). Accordingly, promoters that give rise to Phantom Peaks tend to be highly active (Figure 2D) and show strong activity across different stages of embryonic development when compared to the relevant modENCODE transcriptome profiles (32, data not shown). Curiously, 16% of Phantom Peaks maps to the repressed chromatin in S2 cells, which carries H3K27me3 marks (Figure 2A) (35).

In agreement with their mapping to active promoters, most of the Phantom Peaks co-localize with sites of transcription factors clustering (37). Moreover, Phantom Peaks show considerable overlap with ‘High Occupancy Target’ (HOT) regions (37). These regions have been extracted from the ChIP profiles of 41 sequence-specific *Drosophila* transcription factors (a modENCODE and BDTNP initiative). The analyses identified 1962 HOT genomic regions that are bound by more than 8 and up to 24 different transcription factors. These sites of clustered ChIP signals collectively constitute roughly 3% of the genome. Not all HOT sites have known functions in transcription control and it has been speculated that some may constitute storage hubs for transcription factors. We observed that about one third of the Phantom Peaks overlap with annotated HOT regions (Figure 2E).

### Phantom Peaks are present in ChIP-seq profiles in the modENCODE database

**Figure 3.**
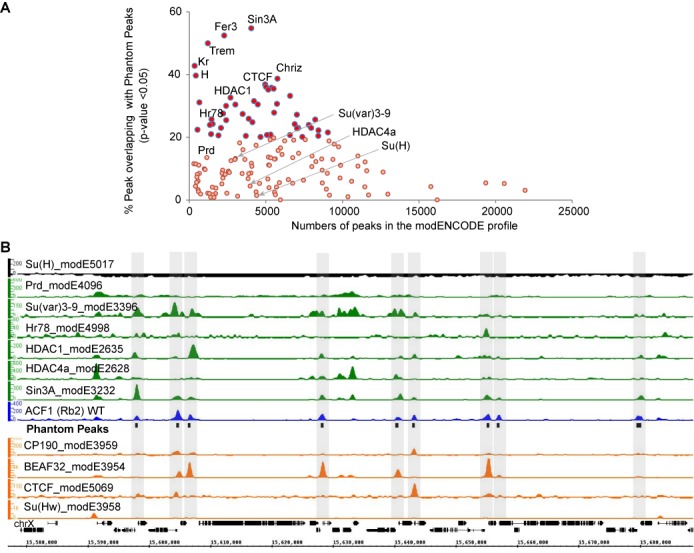
Phantom Peaks coincide with peaks of several modENCODE profiles. (**A**) Peaks called by modENCODE on 153 different non-histone chromatin factors were tested for overlap with Phantom Peak regions. For each profile the scatterplot graph depicts the number of peaks defined by modENCODE and the fraction of these peaks overlapping with Phantom Peaks (*P*-value <0.05). The *P*-values indicate the significance of proximity of the modENCODE profile peaks to the Phantom Peaks. (**B**) Smoothed and background-subtracted tag density profiles of selected modENCODE profiles are shown in a representative genomic region on chromosome X. The positions of Phantom Peaks are indicated by black boxes and gray-shaded rectangles across all profiles.

The chromatin for our ChIP assays was obtained by shearing with adaptive focused acoustics technology (Covaris) to fragments of mean sizes of roughly 200 bp. This method is increasingly used for ChIP (38–42) as it focuses sound waves in the sample in a closed-tube setting and avoids heating. Previously, we employed this method to map male-specific-lethal (MSL) proteins to specific genomics sites that are distinct from Phantom Peaks [e.g. only 10% of MSL2 peaks or 4% of the MLE peaks overlap with Phantom Peaks (*P*-value < 0.05), data not shown (43)]. To explore whether Phantom Peaks are limited to some specific aspects of our methodology, we mined the modENCODE database for ChIP-seq profiles containing signals at Phantom Peak loci. We compared the reported peak sets for 153 non-histone proteins (Supplementary Table S5) with the Phantom Peak regions, requesting an overlap of at least 50 bp. [Similar overlap analysis was performed for 151 histone modification ChIP-seq profiles (Supplementary Table S6)]. The statistical significance of the overlap intervals between two peak sets was calculated using the asymmetric comparison framework outlined by Chikina and Troyanskaya and considering a 4 kilobase (kb) window centered at TSS as reference regions (33). This choice of reference appears appropriate as 92% of Phantom Peaks are localized within such regions. Our analysis for non-histone protein ChIP profiles shows that 31% of the modENCODE profiles consist of peak sets with more than 20% (*P*-value <0.05) of the peaks overlapping with Phantom Peaks (Figure 3; Supplementary Figures S8 and S9). These overlapping peaks are amongst the high scoring peaks, suggesting that they are unlikely to be identified erroneously due to low signal, but enriched during the ChIP procedure (Supplementary Figure S10).

We mapped the raw sequences obtained from modENCODE and used the SPP package to generate background-subtracted smoothened tag density tracks for some profiles that overlap strongly with Phantom Peaks. Among the very similar profiles are the ones for transcription (co-)regulators such as Sin3A, Fer-3, Chriz and Hr78 (55%, 53%, 39%, 24% respective overlap, *P*-value <0.05), histone deacetylases HDAC1 and HDAC6 (31%, 25% respective overlap, *P*-value <0.05) and insulator proteins such as BEAF-32, CTCF and CP190 (36%, 26%, 23% respective overlap, *P*-value <0.05). These overlap percentages probably underestimate the numbers of shared peaks considering the fact that not all potentially enriched regions are called as peaks by the software in both Phantom Peak and modENCODE cases. Similarly processed tracks for weakly overlapping factors such as Prd and Su(H) (13%, 1.4% overlap, *P*-value <0.05) and representatives of the high overlapping factors show similar regions of enrichments of the ChIP signals along the Phantom Peaks in the latter cases (Figure 3B; Supplementary Figures S8 and S9).

Visual inspection reveals a strong correlation between Phantom Peaks and several insulator protein profiles (Figure 3B, Supplementary Figure S8). Indeed, Phantom Peaks show strong coincidence with the ChIP-seq peak sets obtained for CP190, CTCF, BEAF-32 and Su(Hw) from 12 to 14 h old embryo chromatin, (82%, 47%, 81% and 25%, respectively, data not shown). Interestingly, only 9.2%, 22.2% and 24.8% of BEAF-32, CTCF and Su(Hw) peaks contain their known target sequence motif to which these insulators are known to bind. Crosslinking of the respective insulators to the remainder of the sites may be due to targeting principles other than direct DNA binding (44,45) or these peaks may even contain false positive signals as we found for nucleosome remodelers.

DamID offers an antibody-free approach to map chromatin binding sites. In this case, the protein of interest is expressed as in fusion with *E. coli* Dam DNA methylase. The Dam enzyme will be recruited to particular chromatin locations by the fusion partner and methylates DNA in the vicinity, which can be further mapped by chip-hybridization or sequencing (46). Comparison of antibody-generated profiles to DamID profiles may distinguish false positive Phantom Peaks from real signals. If DamID profiles are similar to antibody-generated peaks, the signals are likely to be real, if the overlap is not good, then they could potentially be Phantom Peaks. We compared Phantom Peaks with the reported 219 DamID data sets (27,28) using the same asymmetric comparison framework (33). The peaks in normalized DamID profiles were defined by retrieving the top 1% scoring probe locations from the chip and extending them by 1 kb. We observed 29 profiles corresponding to 16 chromatin proteins (CG4617, CG9797, TIP60, BEAF32B, DMAP1, TBP, CG7928, PHOL, FAIRE, CG10267, CG4936, SIN3A, DWG, MNT, MAX, PCAF) that show 20–40% peak overlap with Phantom Peaks (*P*-value < 0.05). Comparison of antibody-generated modENCODE profiles and DamID profiles with the Phantom Peaks set revealed similarities and differences. The overlap of Sin3A-DamID and BEAF32-DamID with the Phantom Peaks are strong and therefore many sites may indeed be functional. By contrast, DamID profiles of the insulators CTCF and of Su(var)3–9 show poor overlap with Phantom Peaks (see Supplementary Figure S11A,B; Supplementary Table S7), raising doubts about the reliability of the corresponding ChIP-seq profiles. Interestingly, the DamID profile for ISWI (a core component of ACF and RSF complexes) shows poor overlap with Phantom Peaks (about 8% for 2 replicates, *P*-value < 0.05). We further observe that most of the Phantom Peaks are localized within the chromatin derived from housekeeping gene (Supplementary Figure S11C).

## DISCUSSION

ChIP-seq profiles are commonly assumed valid if the data have been generated in concert with the modENCODE guidelines that were drafted to assure a good quality of data entered in public repositories. These guidelines demand that researchers validate the specificity of their antibodies by Western blotting and immunofluorescence microscopy (if possible) and that two independent antibodies against a chromatin protein yield consistent results (47). Our ChIP-seq reagents meet these requirements. Using four different antibodies directed against two signature subunits of remodeling factors, we derived ChIP-seq profiles with excellent signal to noise ratios identifying 3000 enriched genomic loci. However, repeating the analysis with two different fly mutants lacking the antigens, we obtained the same profiles, leading us to conclude that the profiles do not reflect *bona fide* binding sites for the remodelers. This additional specificity control is not commonly included in ChIP-seq analyses because suitable mutants are sometimes not available and knockdown strategies are often too inefficient to remove the antigen completely.

Although the enrichment of Phantom Peaks is non-specific from the antibody point of view, the genomic sites that are retrieved are by far not random. The majority of Phantom Peaks maps to promoters of actively transcribed genes that are characterized by open chromatin and are bound by multiple transcription factors and the transcription machinery. A large fraction of Phantom Peaks also coincides with previously mapped HOT regions, where between 8 and 24 transcription factors have been found co-localizing.

We speculate that the enrichment of these sites is brought about by a combination of poor ChIP specificity and interaction-prone (‘sticky’) surfaces at the Phantom Peak locations. Phantom Peaks are unlike to be retrieved if the specific ChIP reaction is efficient, like in our earlier experiments on the MSL proteins (43). In the case of the remodeling enzymes this was apparently not the case. Although our antibodies are able to immunoprecipitate the antigens efficiently, their target proteins are apparently not well cross-linked to chromatin, due to their dynamic and transient chromatin interactions. For ISWI-containing remodeling factors fluorescence-based measurements of their dynamic mobility in live cells have revealed that only a minor fraction (<3%) of them actually engage with the nucleosomal substrates. This fraction may increase under certain circumstances, for example in the context of chromatin assembly during replication or repair, but in these cases binding events will be delocalized and will not be represented as peaks (15,16,48). In the absence of such specific cues, formaldehyde crosslinking may trap highly mobile proteins to the most accessible sites in the genome. However, such a scenario does not explain the presence of peaks in the absence of the antigen.

Why should regions of high transcription factor occupancy and recruitment of the RNA polymerase machinery be prone to non-specifically interact with antibodies? We speculate that many transcription factors and components of the transcription machinery contain ‘unstructured’ protein surfaces or molten-globule conformations, sometimes of low sequence complexity or highly charged, that are poised to interact with close-by targets and to acquire a specific structure only through this interaction (49–52). For example, low complexity protein domains of some transcription factors have the potential to interact with the equally unstructured CTD of RNA polymerase II (53). Conceivably, such unstructured domains may interact with antibodies non-specifically, once their native assembly has been disrupted by sonication. In such a hypothetical scenario, sites where proteins accumulate bearing interaction-prone surfaces, such as transcription activators, may manifest as Phantom Peaks. Similar local amassing of proteins may also occur at replication forks or sites of DNA repair, but these would not appear as defined peak in ChIP experiments.

We found that a considerable number of modENCODE ChIP-seq profiles display enrichments that coincide with Phantom Peaks. It is currently not possible to evaluate whether these signals reflect true chromatin binding events or other examples of Phantom Peak manifestation. Conceivably, many profiles may be composites of *bona fide* binding sites and Phantom Peaks, with varying contributions of each group. Undoubtedly many published interactions at Phantom Peak sites will prove correct—after all, many of these are highly active promoters, where numerous regulators are known to accumulate. However, in cases where no consensus binding sequences for a sequence-specific factor can be found at the mapped site (as is the case for many insulator proteins), or the functional significance of presumed interactions is unclear (as in the case of HDAC complexes at highly active promoters), or when the massive enrichment of many factors at some sites does not make functional sense (as in the case of HOT regions), caution should be taken.

Our study echoes related observations in other systems. Hyper-ChIPable regions at accessible genomic regions have previously been described in bacteria, yeast and mammals that can be retrieved using antisera directed against non-chromatin proteins (54–57) demonstrating that the problem is not limited to *Drosophila*. False positive signals that are obvious from irrelevant antibodies (e.g. GFP) or pre-immune sera can be reduced by appropriate normalization procedures (3,54). Crosslinking artifacts can be avoided by profiling proteins under native conditions (58), or employing antibody-free methods such as Calling Card-seq or DamID (46,59). These methods have their own specific shortcomings, but offer another viewpoint to define genomic targets.

Concerning some shortcomings of antibodies, such as relaxed specificity and cross-reactivity, the increasing awareness in the research community has led to initiatives toward increased standardization and quality control measures (60). For antibody-mediated ChIP the false positive signals can only be revealed if knockout or knockdown controls are implemented. When such controls are difficult to obtain, our list of Phantom Peaks may serve as a catalog of sites, whose noticeable enrichment in the ChIP profiles should raise a ‘red flag’ and demand additional verification before making any functional inferences. The *Drosophila* genomics community will profit from a listing of such dubious sites, just as the proteomics community has profited from publication of a listing of the cRAP protein sequences of common contaminants in mass spectrometric analyses (61).

## Supplementary Material

SUPPLEMENTARY DATA
