# Supplementary material for: Active promoters give rise to false positive ‘Phantom Peaks’ in ChIP-seq experiments
Source: Nucleic Acids Res. 2015 Jun 27;43(14):6959–68. doi: 10.1093/nar/gkv637 (PMC4538825; doi:10.1093/nar/gkv637)
Supplement: SUPPLEMENTARY DATA [file supp_43_14_6959__index.html]

Active promoters give rise to false positive ‘Phantom Peaks’ in ChIP-seq experiments — SUPPLEMENTARY DATA 

# Active promoters give rise to false positive ‘Phantom Peaks’ in ChIP-seq experiments

## SUPPLEMENTARY DATA

- SUPPLEMENTARY DATA
- SUPPLEMENTARY DATA
- SUPPLEMENTARY DATA
- SUPPLEMENTARY DATA
- SUPPLEMENTARY DATA
- SUPPLEMENTARY DATA
