# Supplementary material for: Active promoters give rise to false positive ‘Phantom Peaks’ in ChIP-seq experiments
Source: Nucleic Acids Res. 2015 Jun 27;43(14):6959–68. doi: 10.1093/nar/gkv637 (PMC4538825; doi:10.1093/nar/gkv637)
Supplement: SUPPLEMENTARY DATA [file supp_gkv637_gkv637__Supplementary_Information_Jain_Becker.pdf]

Primer name	Sequence
Intron9, Timeout_F	agtcatgtgcgacatttgg
Intron9, Timeout_R	acgcaacaagaatggagagg
3' of Fz2_F	cgtactgtcaactgcttg
3' of Fz2_R	cagttctggaataccatgtc
Intron3, Spn_F	ctgttgttgggcagacttagc
Intron3, Spn_R	attggttcagctggttcgtg
3' of CG6465_F	tgtcctcgctttagcttagcc
3' of CG6465_R	tggtgcgcgtcactactatg
5' T48 and Ro_F	gtcttcatacaagcagtgtgtgc
5' T48 and Ro_R	aggttgttgtcctcgcaaag

Supplementary Table 1: List of quantitative PCR primers

ChIP	Input
ACF1-3F1 WT rep1	Input WT Chromatin1
ACF1-3F1 WT rep2	Input WT Chromatin2
ACF1-3F1 WT rep3	Input WT Chromatin3
ACF1-Rb1 acf1 rep1	Input acf1 Chromatin1
ACF1-Rb2 acf1rep1	Input acf1Chromatin1
ACF1-Rb2 acf1rep2	Input acf1 Chromatin2
ACF1-Rb1 WT rep1	Input WT Chromatin4
ACF1-Rb2 WT rep1	Input WT Chromatin4
ACF1-Rb2 WT rep2	Input WT Chromatin5
ACF1-Rb2 WT rep3	Input WT Chromatin6
ACF1-Preimmune-Rb1 WT rep1	Input WT Chromatin5
ACF1-Preimmune-Rb2 WT rep1	Input WT Chromatin4
ACF1-Preimmune-Rb2 WT rep2	Input WT Chromatin5
RSF-1-Rb rsf-1 rep1	Input rsf1 Chromatin1
RSF-1-Rb WT rep1	Input WT Chromatin7
RSF-1-Rb WT rep2	Input WT Chromatin4

Supplementary Table 2: Profiles used for ChIP-seq analysis and their respective input control.

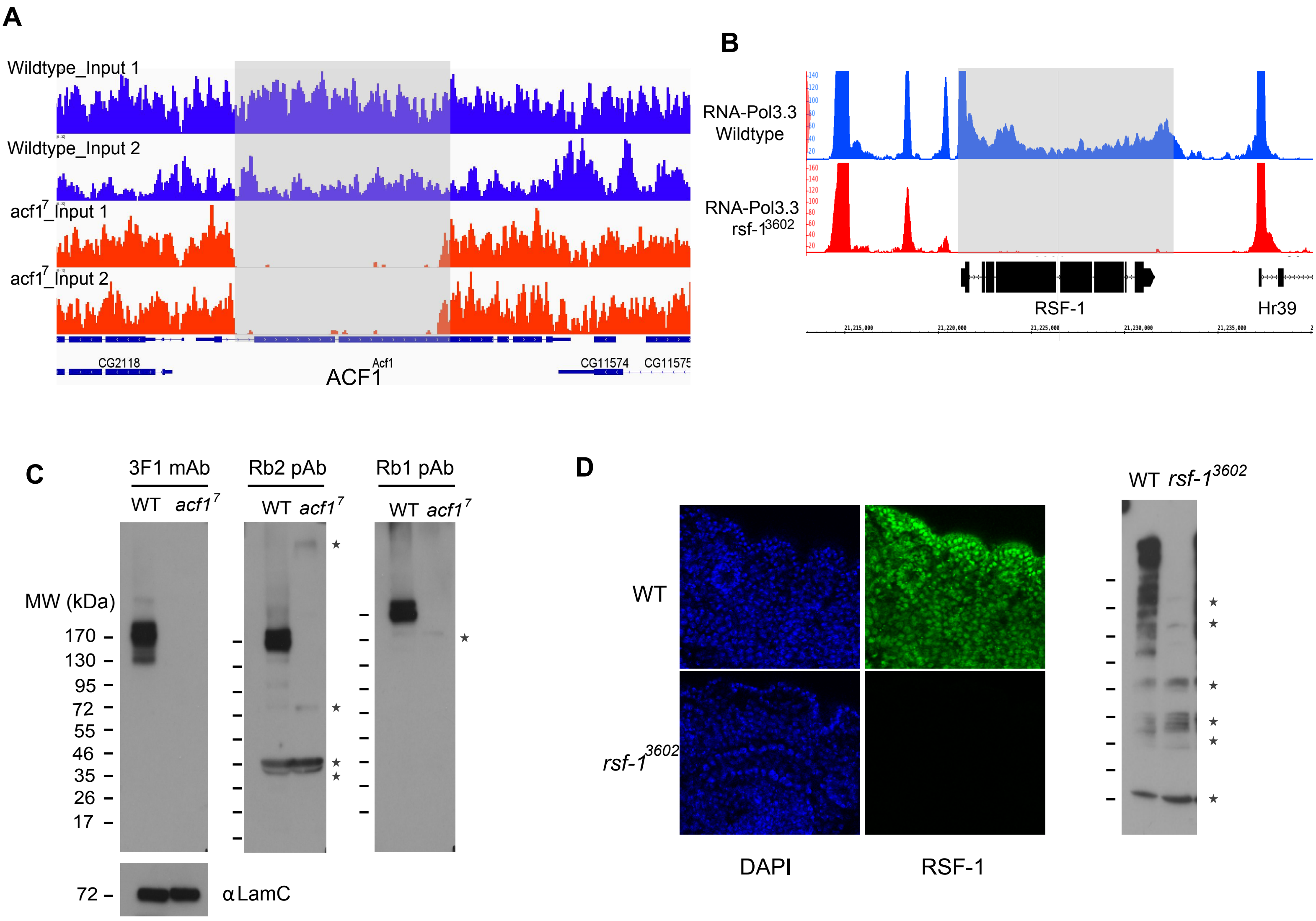

Figure S1: Characterization of mutant embryo chromatin and antibodies.

(A) The *acf1*^{7} allele bears a deletion of ~3.5 kb from the 5' end of the gene that had been generated by imprecise excision of a P-element. The deletion is obvious in ChIP-seq traces of input chromatin of mutant (red) versus wild-type (blue) embryos. Raw read densities are displayed.

(B) In *rsf-1*^{3602} part of the 5' region of the RSF-1 gene is deleted abolishing transcription of the gene as seen by the absence of ChIP-seq signal of RNA polymerase II (unpublished). Raw read densities are displayed.

(C) Western blot analysis of extracts from wild-type and *acf1*^{7} mutant embryos using three different antibodies directed against ACF1.

(D) Left panel: IFM visualization of RSF-1 in nuclei of wild-type and *rsf-1*^{3602} embryos. Right panel: Western blot analysis of extracts from wild-type and *rsf-1*^{3602} embryos.

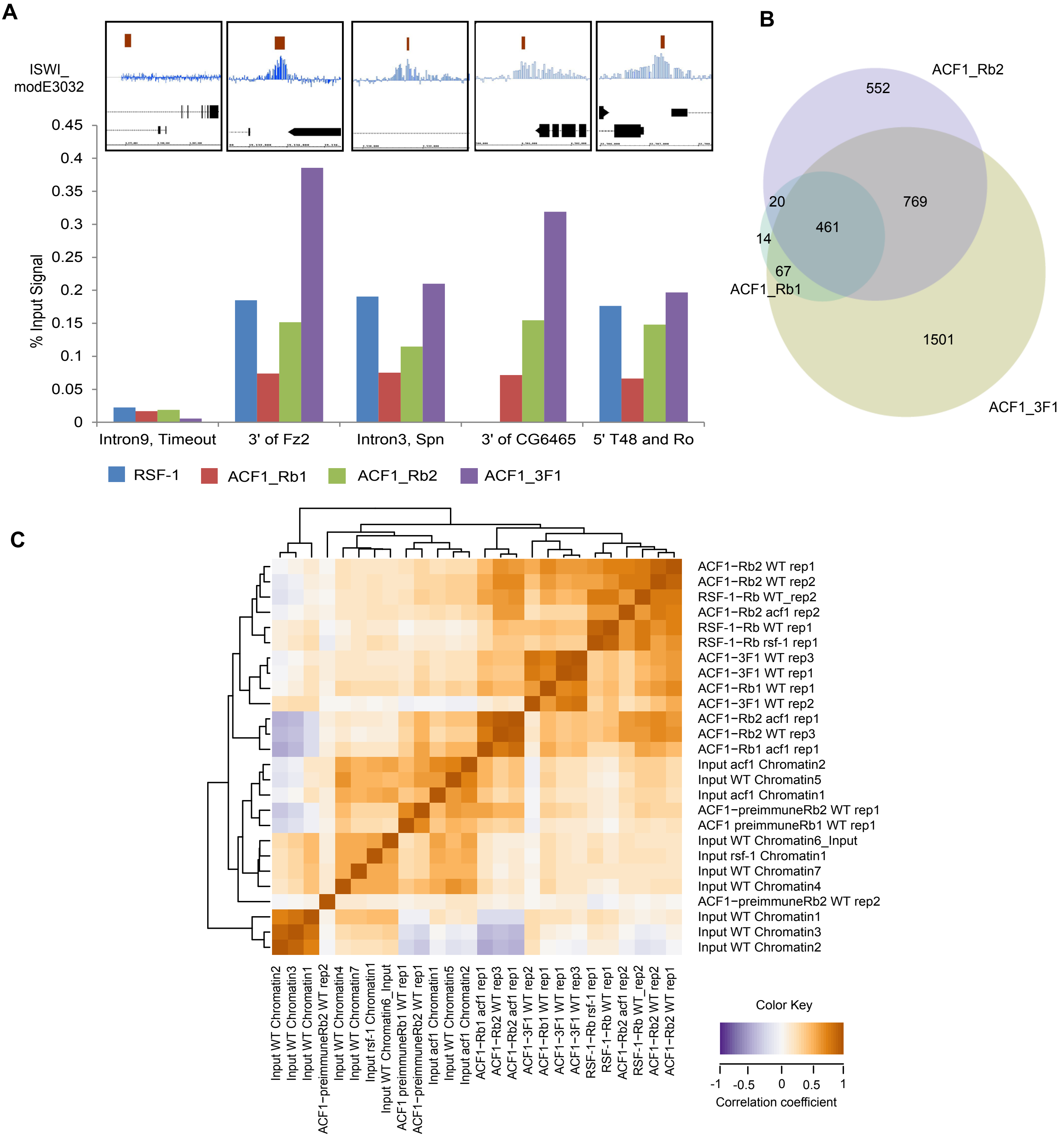

Figure S2: Comparison of ChIP-Seq peak profiles

(A) Immunoprecipitation with all antibodies enrich genomic candidate ISWI binding sites suggested from modENCODE ISWI profiles -mapping to the Fz2, CG6465, Ro/T48 loci. Intron 9 of timeout serves as a negative control region. For each amplicon the corresponding ChIP-chip profile of ISWI as provided by modENCODE is shown on the top. The position of the amplicon is indicated by a red box. Bottom: ChIP-qPCR analysis using the indicated antibodies. Enrichments are given in percent of the input values.

(B) Venn diagram showing the overlap of peaks defined by ChIP-seq using three different ACF1 antibodies.

(C) Correlation between the ChIP-seq profiles. The size-normalized sequence tags were counted for all 16 ChIP and 10 input samples in the Phantom Peak regions. The matrix illustrates the degree of similarity obtained by Spearman rank-based correlation. The heat map displays the correlation coefficients for all pairwise comparisons.

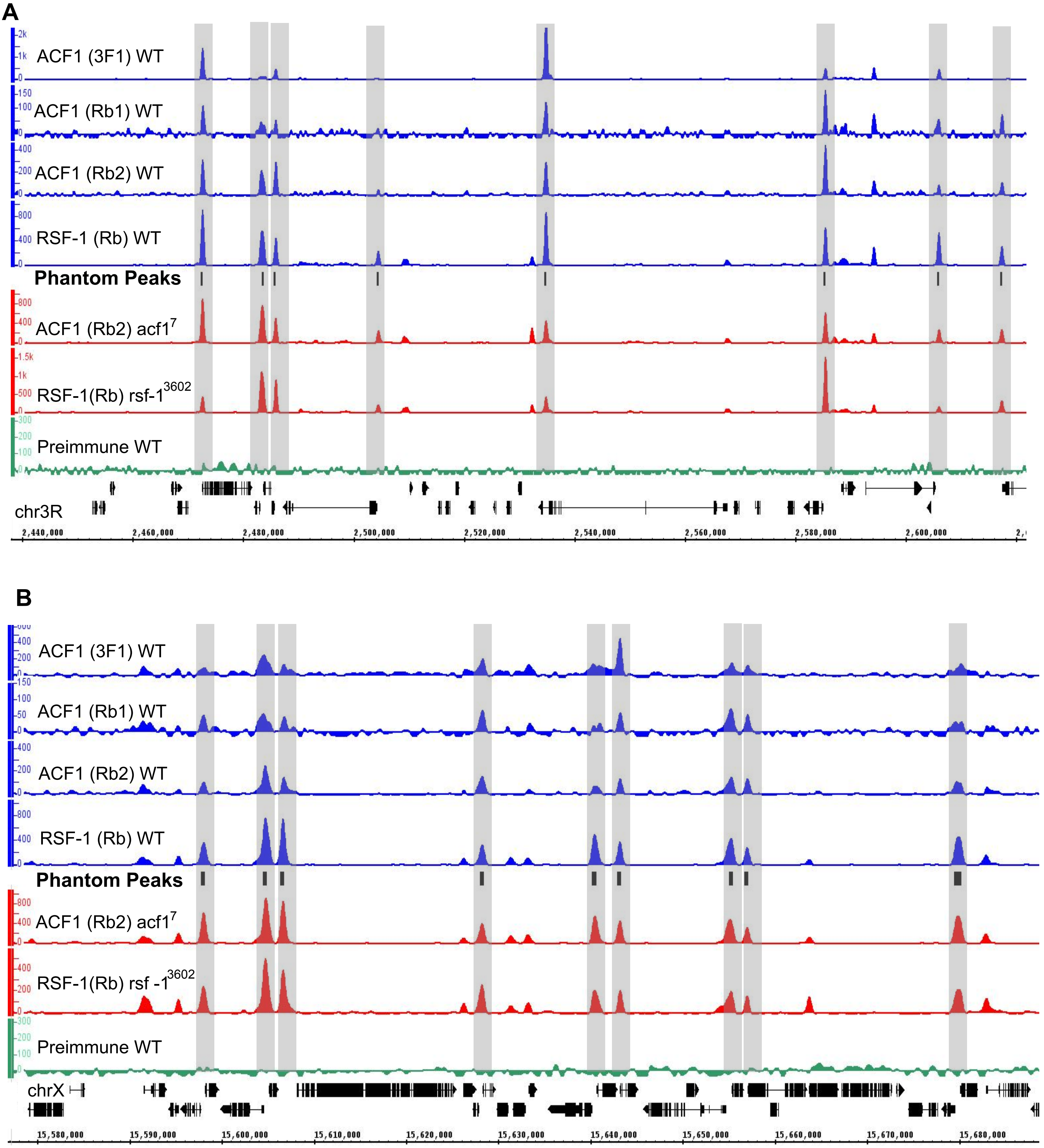

Figure S3: Snapshots of the remodeling factor ChIP-seq profiles.
Both panels show representative genome browser snapshots of the smoothened and back-ground-subtracted tag densities for ACF1 and RSF-1 ChIP-seq profiles, similar to Figure 1. The upper panel displays a genomic region from chromosome 3R, the lower one is derived from the X chromosome.

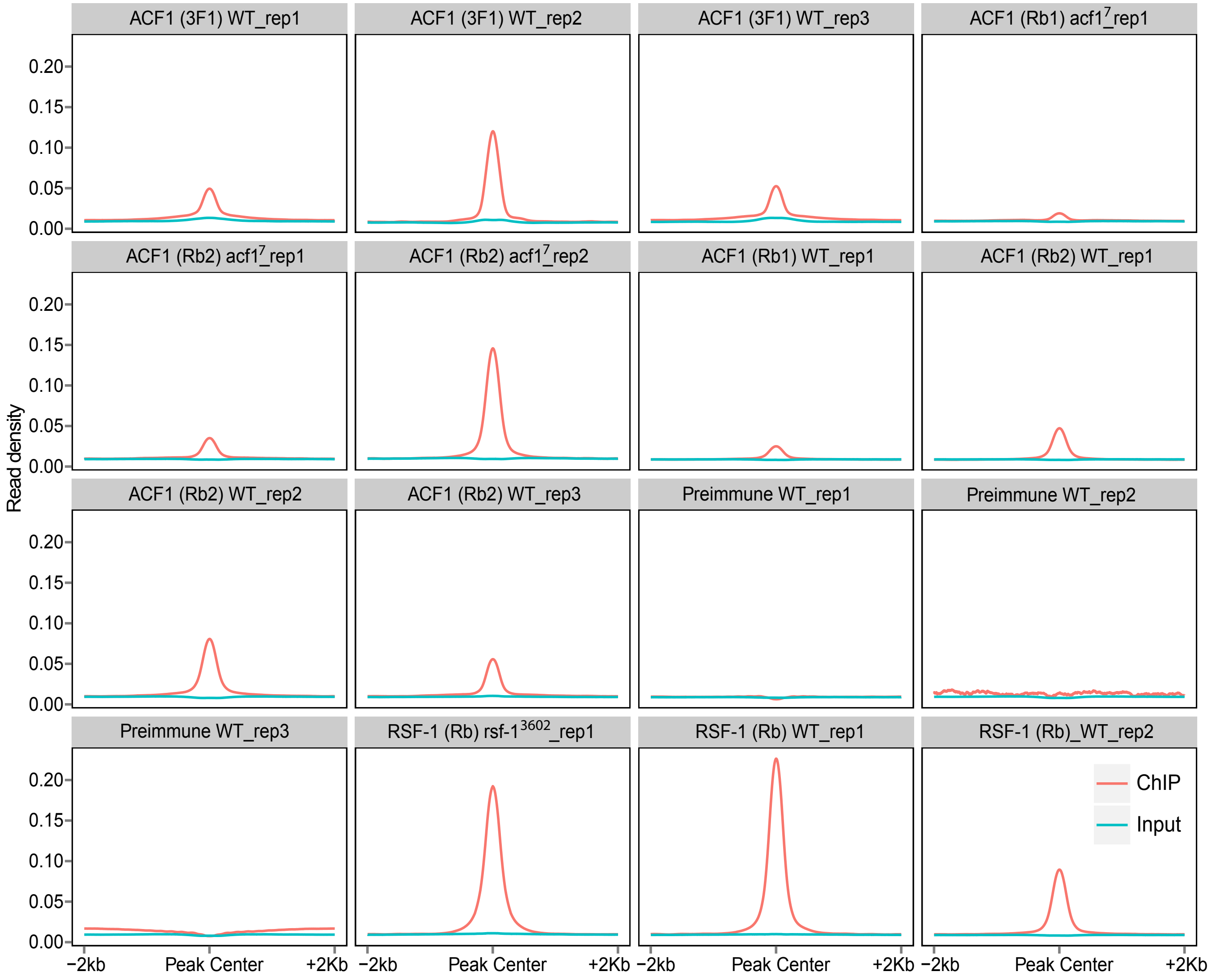

Figure S4: Phantom Peak regions show specific enrichment in the ChIP samples.
Library size-normalized reads are plotted along all 3090 Phantom Peak regions for the ChIP and corresponding input control samples. The header includes immunoprecipitated protein, the antibody, the corresponding genotype and the replicate.

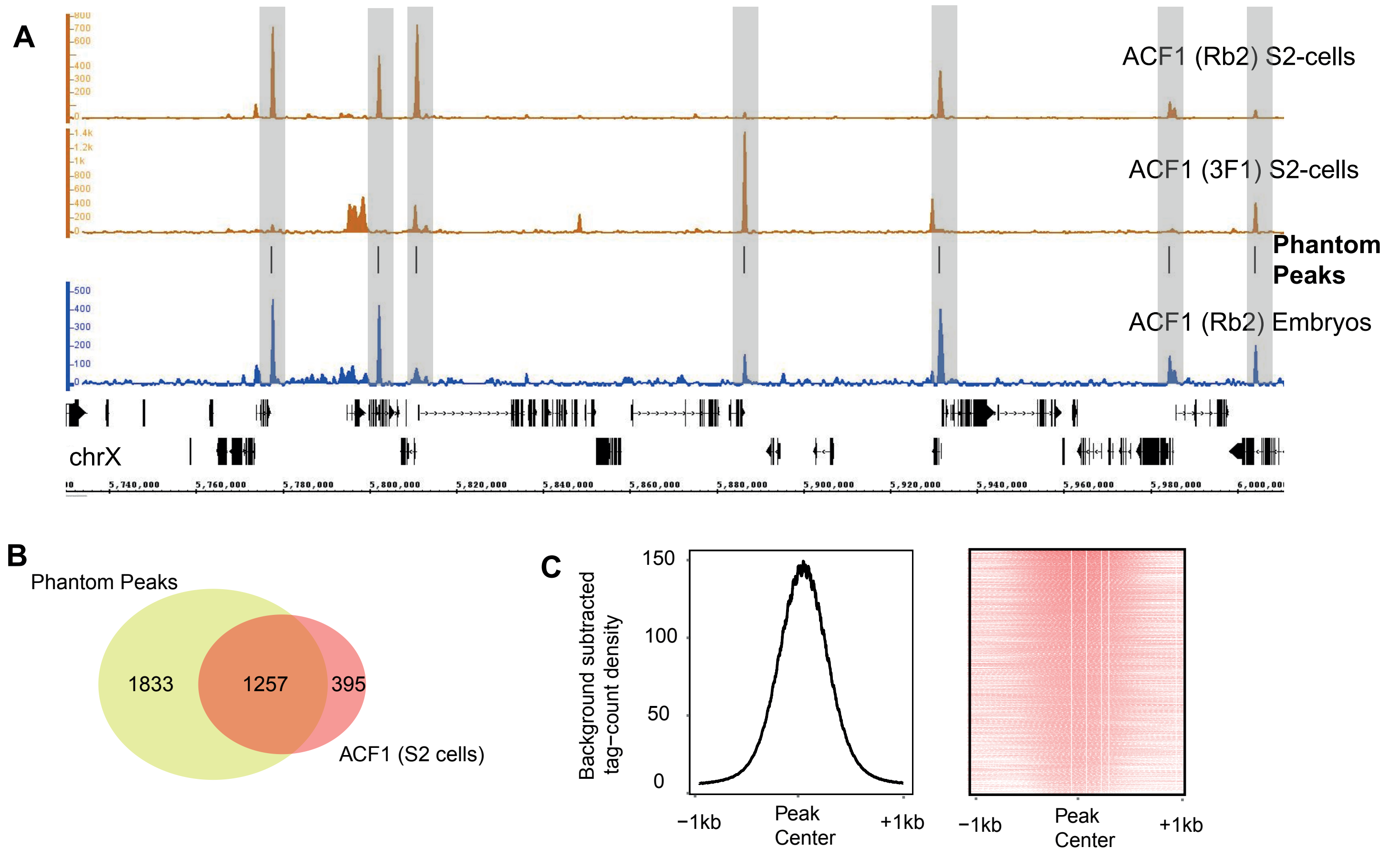

Figure S5: Phantom Peaks can be identified upon lowering the formaldehyde concentration from S2 cells.

(A) Background tag subtracted smoothened tracks show ACF1 ChIP-seq profiles from 1% FA fixed S2 cells using two antibodies. Enrichment along the Phantom Peaks is visible.

(B) Peaks identified in S2 cells strongly overlap with Phantom Peaks. (C) Cumulative density plot and heat-map representation of the ACF1 ChIP-Seq signals along Phantom Peaks in combined S2 cell profiles. The log transformed signal in the heat-map appears quantized due the use of low resolution input normalized ChIP-seq wiggle files calculated by SPP package.

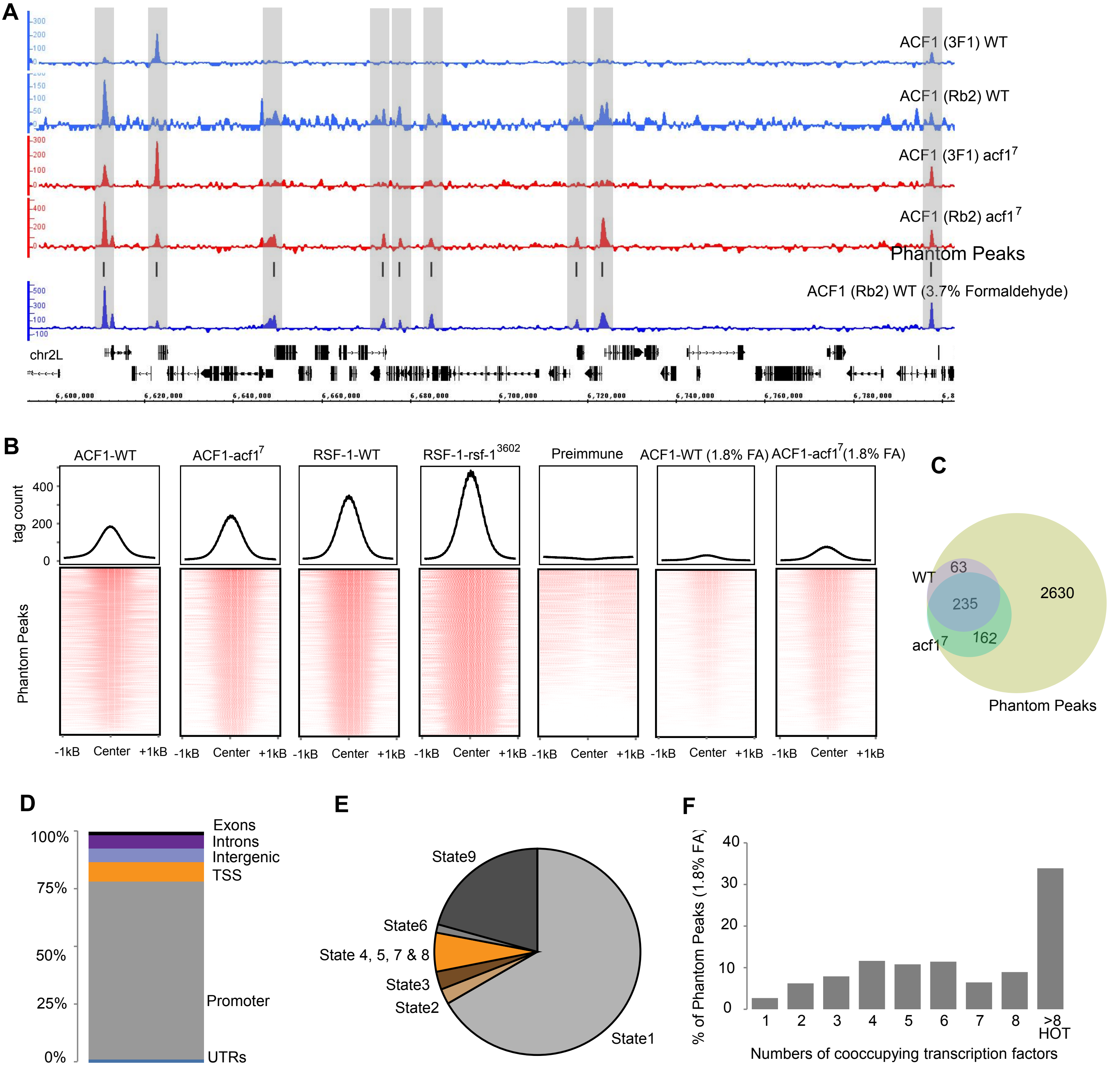

Figure S6: Comparison of ChIP-Seq profiles obtained using 1.8% formaldehyde (FA). ACF1 ChIP-Seq analysis was performed using 3F1 and Rb2 antibodies on 0-12 hour old embryos of WT and acf1^7 genotype, where chromatin was fixed with 1.8% FA.

(A) Background-subtracted tag density profiles in WT (blue) and acf1^7 (red). For comparison, the Rb2 ACF1 ChIP-profile obtained using 3.7% FA is juxtaposed.

(B) The ChIP-Seq signals along the previously identified 3090 Phantom Peaks are displayed as cumulative density plots and heatmaps for the different genotypes or crosslinking conditions (3.7% FA unless indicated differently). The log transformed signal in the heat-map appears quantized due the use of low resolution input normalized ChIP-seq wiggle files calculated by SPP package

(C) Overlap analysis of peaks identified in 1.8% formaldehyde fixed WT and acf1^7 embryos with the Phantom Peaks.

(D) Genomic feature annotation, (E) chromatin state assignment and (F) transcription factor co-localization along the “Phantom Peaks (1.8% FA)” are displayed.

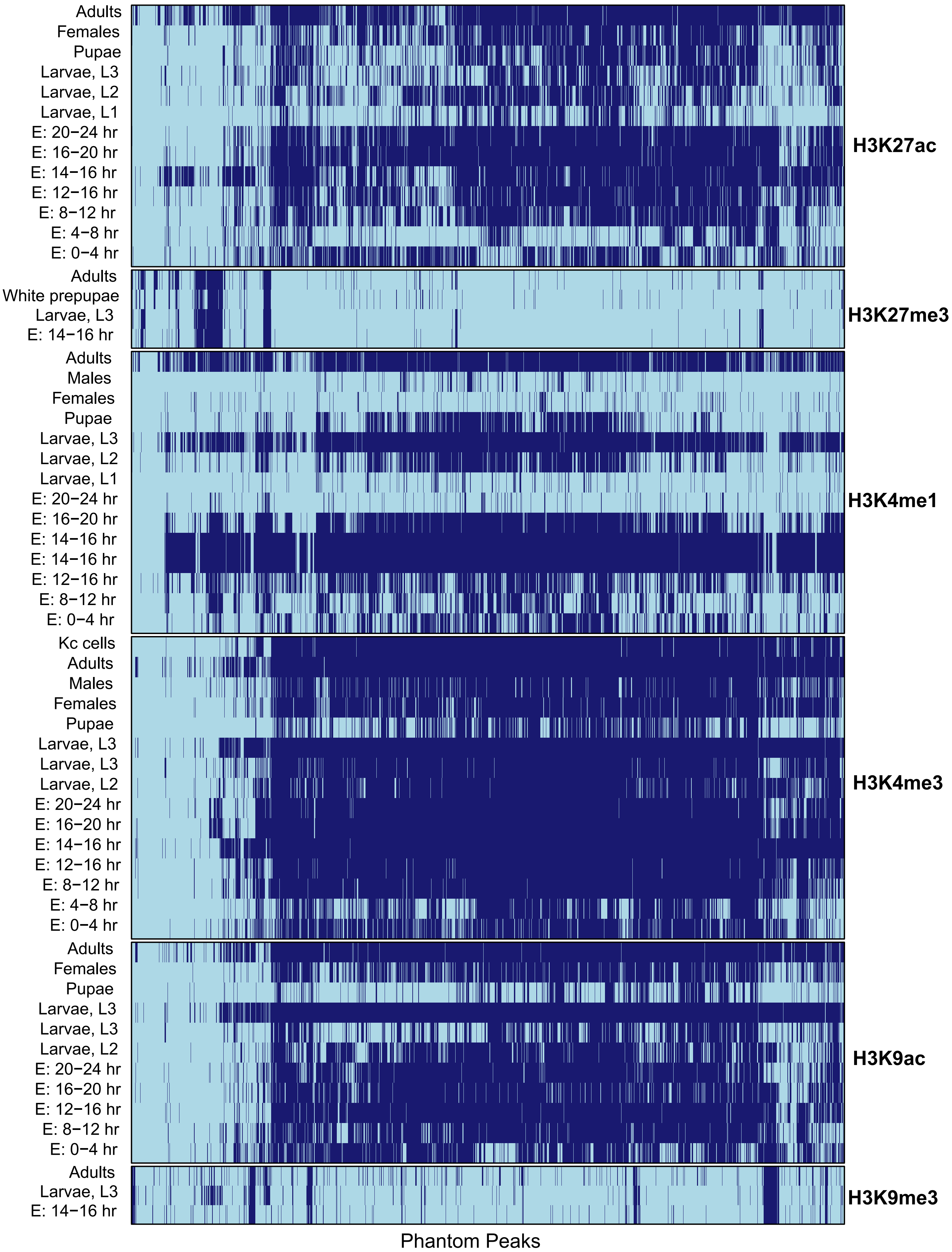

Figure S7: The Phantom Peak regions tend to harbor active chromatin marks during different stages of development. Phantom Peak regions were mapped across active (H3K4me1/me3, K9ac, K27ac) and re-pressive (H3K9me3/ 27me3) histone modification profiles deposited in the modENCODE resource. Binary scoring of the presence of a mark along the investigated site was used to generate the heatmap. Dark blue represents overlap with the histone mark.

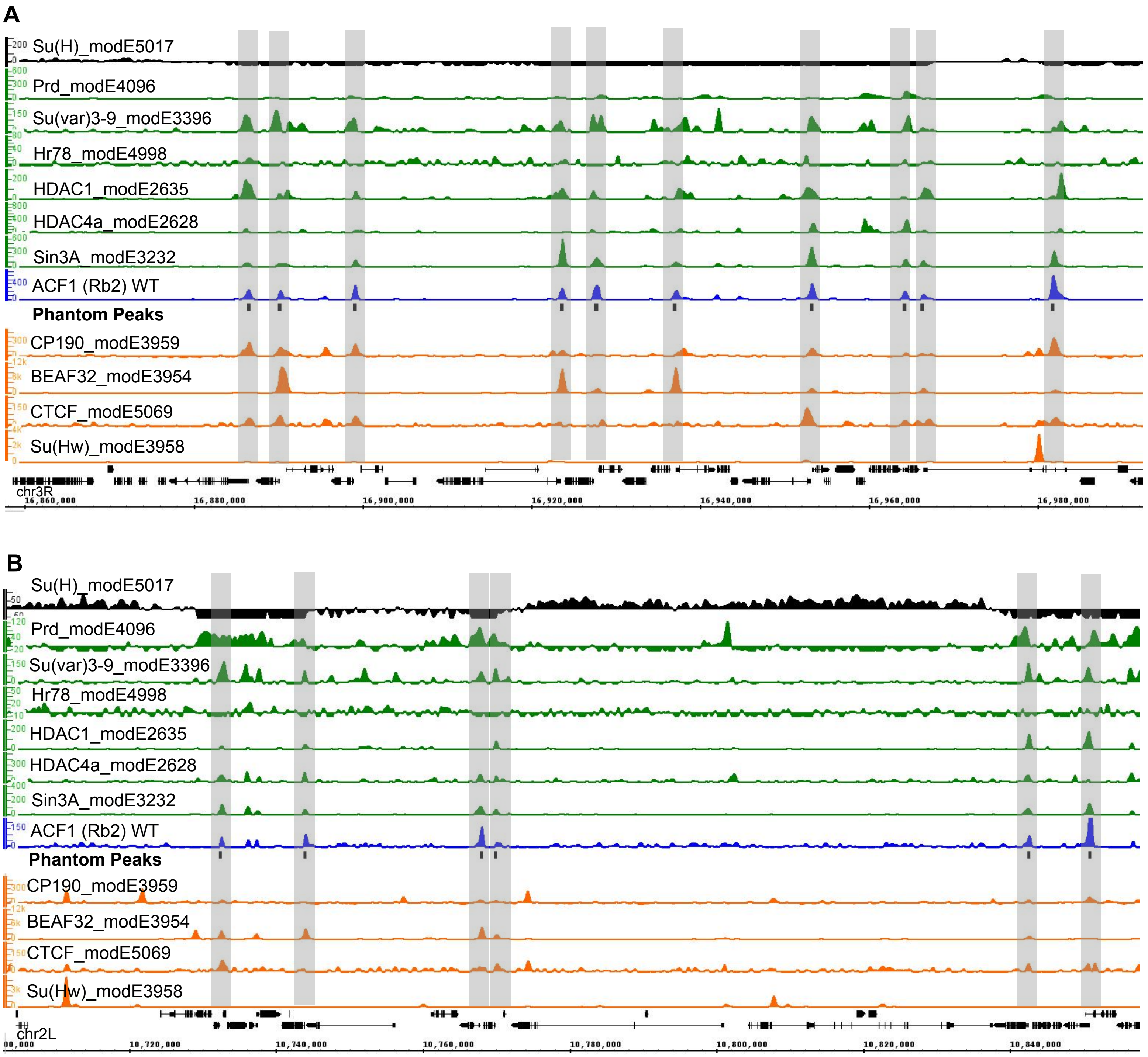

Figure S8: Snapshot of modENCODE profiles along the Phantom Peaks.
Smoothed and background-subtracted tag density profiles of selected modENCODE profiles are shown in a representative genomic region on chromosome 3R (A) and 2L (B). The positions of Phantom Peaks are indicated by black boxes and grey-shaded rectangles across all profiles.

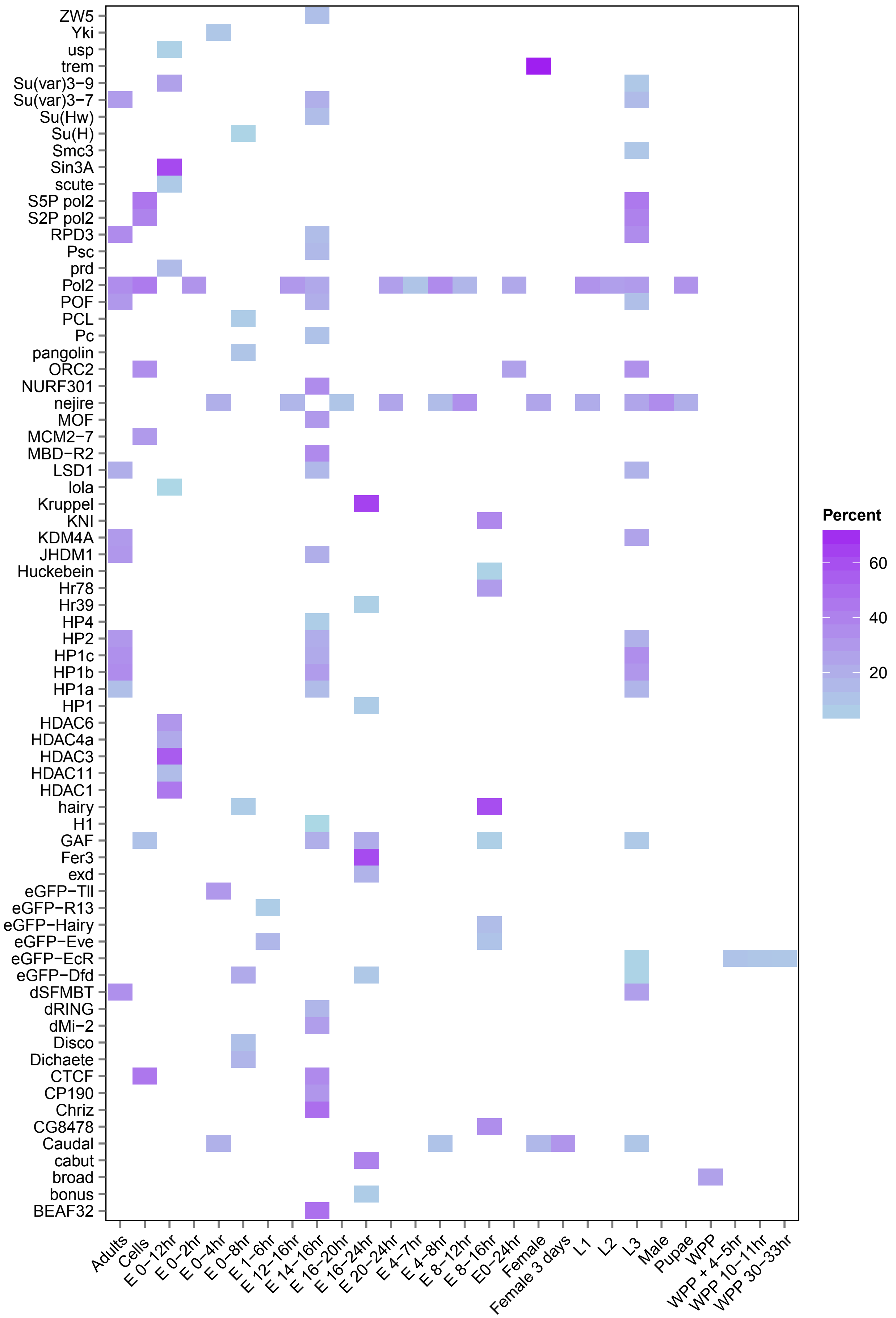

Figure S9: modENCODE profile overlaps with the Phantom Peaks**.**
Heatmap displaying the percent overlap of all modENCODE profile peaks with Phantom Peak regions. The ChIP targets are organized in rows, the developmental stages in columns.

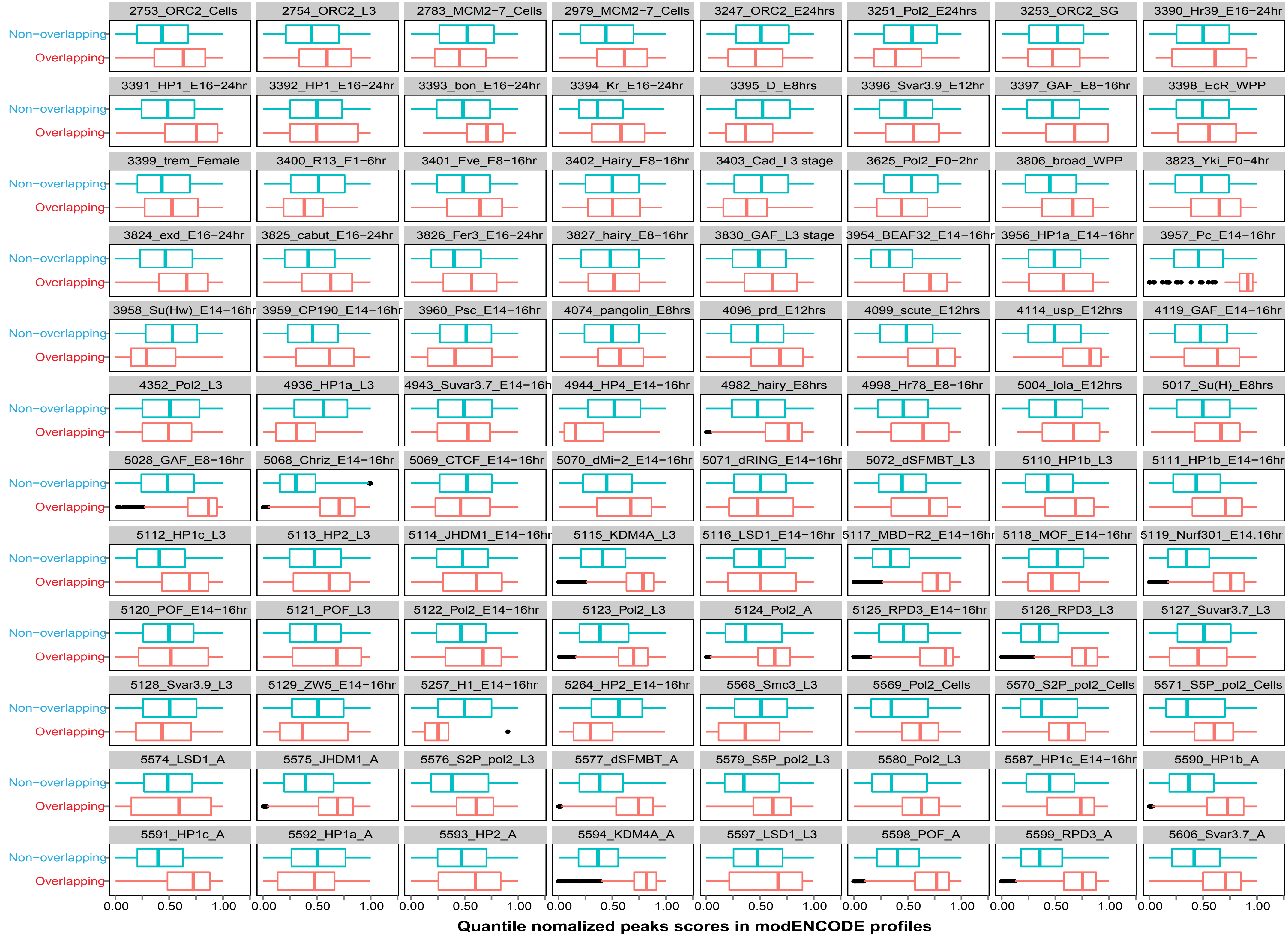

Non−overlapping
Overlapping
2753_ORC2_Cells
2754_ORC2_L3
2783_MCM2−7_Cells
2979_MCM2−7_Cells
3247_ORC2_E24hrs
3251_Pol2_E24hrs
3253_ORC2_SG
3390_Hr39_E16−24hr
3391_HP1_E16−24hr
3392_HP1_E16−24hr
3393_bon_E16−24hr
3394_Kr_E16−24hr
3395_D_E8hrs
3396_Svar3.9_E12hr
3397_GAF_E8−16hr
3398_EcR_WPP
3399_trem_Female
3400_R13_E1−6hr
3401_Eve_E8−16hr
3402_Hairy_E8−16hr
3403_Cad_L3 stage
3625_Pol2_E0−2hr
3806_broad_WPP
3823_Yki_E0−4hr
3824_exd_E16−24hr
3825_cabut_E16−24hr
3826_Fer3_E16−24hr
3827_hairy_E8−16hr
3830_GAF_L3 stage
3954_BEAF32_E14−16hr
3956_HP1a_E14−16hr
3957_Pc_E14−16hr
3958_Su(Hw)_E14−16hr
3959_CP190_E14−16hr
3960_Psc_E14−16hr
4074_pangolin_E8hrs
4096_prd_E12hrs
4099_scute_E12hrs
4114_usp_E12hrs
4119_GAF_E14−16hr
4352_Pol2_L3
4936_HP1a_L3
4943_Suvar3.7_E14−16h
4944_HP4_E14−16hr
4982_hairy_E8hrs
4998_Hr78_E8−16hr
5004_lola_E12hrs
5017_Su(H)_E8hrs
5028_GAF_E8−16hr
5068_Chriz_E14−16hr
5069_CTCF_E14−16hr
5070_dMi−2_E14−16hr
5071_dRING_E14−16hr
5072_dSFMBT_L3
5110_HP1b_L3
5111_HP1b_E14−16hr
5112_HP1c_L3
5113_HP2_L3
5114_JHDM1_E14−16hr
5115_KDM4A_L3
5116_LSD1_E14−16hr
5117_MBD−R2_E14−16hr
5118_MOF_E14−16hr
5119_Nurf301_E14.16hr
5120_POF_E14−16hr
5121_POF_L3
5122_Pol2_E14−16hr
5123_Pol2_L3
5124_Pol2_A
5125_RPD3_E14−16hr
5126_RPD3_L3
5127_Suvar3.7_L3
5128_Svar3.9_L3
5129_ZW5_E14−16hr
5257_H1_E14−16hr
5264_HP2_E14−16hr
5568_Smc3_L3
5569_Pol2_Cells
5570_S2P_pol2_Cells
5571_S5P_pol2_Cells
5574_LSD1_A
5575_JHDM1_A
5576_S2P_pol2_L3
5577_dSFMBT_A
5579_S5P_pol2_L3
5580_Pol2_L3
5587_HP1c_E14−16hr
5590_HP1b_A
5591_HP1c_A
5592_HP1a_A
5593_HP2_A
5594_KDM4A_A
5597_LSD1_L3
5598_POF_A
5599_RPD3_A
5606_Svar3.7_A
0.00
0.50
1.00
Quantile nomalized peaks scores in modENCODE profiles

Figure S10: The modENCODE peaks overlapping with Phantom Peaks seem to have higher calculated peak strengths.
We selected 96 non-histone modENCODE ChIPseq profiles for which peak scores were provided. The scores were quantile-normalized for each profile and partitioned into set overlapping or not with Phantom Peaks. Both sets are plotted as boxplots. Panel headers indicate modENCODE accession number, target and developmental stage.

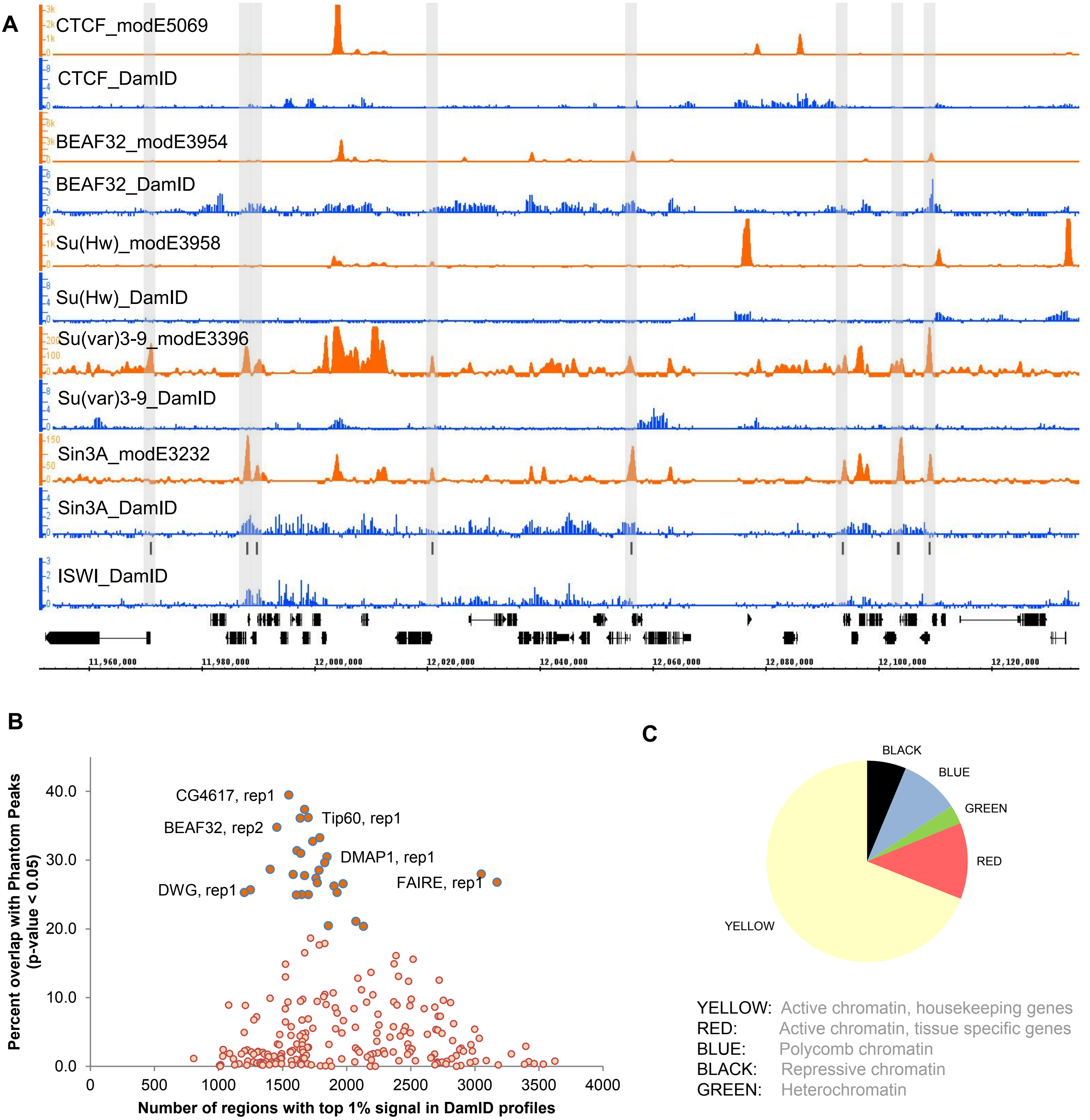

Figure S11: Several DamID profiles show poor overlaps with Phantom Peaks.
(A) Background tag subtracted smoothened modENCODE ChIP profiles are juxtaposed to the corresponding DamID profile for the selected factors. DamID profiles represents Dam only normalized signal.
(B) Peak regions identified on DamID profiles were scored for their overlaps with the Phantom Peaks. Percent overlapping peaks at p-value cutoff of 0.05 are plotted against the total numbers of DamID peaks
(C) Distribution of the Phantom Peaks along the 5-state chromatin model built using DamID profiles.
